# Cholesterol and Inflammation in Atherosclerosis: An Immune-Metabolic Hypothesis

**DOI:** 10.3390/nu12082444

**Published:** 2020-08-14

**Authors:** Didac Mauricio, Esmeralda Castelblanco, Nuria Alonso

**Affiliations:** 1 Department of Endocrinology & Nutrition, Hospital de la Santa Creu i Sant Pau & Institut d’Investigació Biomédica Sant Pau (IIB Sant Pau), 08041 Barcelona, Spain; esmeraldacas@gmail.com; 2 Centre for Biomedical Research on Diabetes and Associated Metabolic Diseases (CIBERDEM), Instituto de Salud Carlos III, 08907 Barcelona, Spain; nalonso32416@yahoo.es; 3 Faculty of Medicine, University of Vic—Central University of Catalonia (UVic/UCC), 08500 Vic, Spain; 4 Department of Endocrinology & Nutrition, University Hospital & Health Sciences Research Institute Germans Trias i Pujol, 08916 Badalona, Spain; 5 Department of Medicine, Barcelona Autonomous University (UAB), 08916 Badalona, Spain

Atherosclerotic cardiovascular disease (ASCVD) is a major cause of morbidity and mortality worldwide [1]. Societal and technological advances during the past few decades in both developed and developing countries have positively impacted the health and well-being of the population and ultimately translated into improved life expectancy and quality of life. However, this so-called progress has led to a sharp increase in the incidence of several conditions that are considered important risk factors for atherosclerosis—mainly obesity, metabolic syndrome, type 2 diabetes mellitus, hypertension, and dyslipidemia. The reasons behind this increase include changes in people’s lifestyles and preferences—in particular, unhealthy food consumption and decreased physical activity.

An excellent review by Tsoupras et al., published in *Nutrients* a couple of years ago had a strong impact on the readership along with a large number of citations [2]. In this piece of work, the authors provided a challenging view on the primary role of inflammation in the pathogenesis of several chronic conditions, including ASCVD. Indeed, the authors’ position in the paper supported the notion that inflammation is the cause of several chronic diseases, such as cancer; some cerebral disorders; asthma; and, notably, atherosclerosis. In support of this contention, the authors performed a thorough review of the literature, including their own work, with a special focus on the role of a key inflammatory molecule—namely, platelet-activated factor. For the interested reader, this is certainly a nice piece of work that brought in a challenging and groundbreaking perspective on this matter. It is out of the scope of the current editorial to address all the issues raised in this lengthy review paper. We are just aiming hereby to provide a complementary view of the controversy raised by the authors regarding the role of lipids and inflammation in the pathogenesis of atherosclerotic disease.

From the title to its conclusions, the paper by Tsoupras et al. offered a different perspective on the causative role of the inflammatory response in the pathogenesis of atherosclerosis, as opposed to what they define as the traditional “lipid hypothesis”. This line of thought is really interesting to the field, both from the perspective of the study of the pathophysiology of atherosclerosis as well as from that of the future development of new therapeutic strategies for ASCVD. Tsoupras et al. highlighted that inflammation has a causative role in the onset and progression of atherosclerosis. They also pointed out that healthy lifestyle patterns, especially the Mediterranean diet, have a significant role in the prevention of inflammatory changes leading to atherosclerosis. Indeed, the PREDIMED trial demonstrated that the Mediterranean diet prevented cardiovascular (CV) events, along with the decrease in inflammatory biomarkers [3].

We want to provide here some further thoughts that may help to delineate the complex pathogenetic mechanisms of atherosclerosis and the therapeutic implications derived from their proper characterization. It is well established that atherosclerosis is an inflammatory disease with an important contribution to the immune system in different stages of the process [4,5]. The initiation of this process involves endothelial dysfunction with the subendothelial deposition of modified lipoproteins, which are key for immune activation and the induction of vascular wall inflammation. Thus, both lipoprotein metabolism and the inflammatory immune response play crucial roles in the initiation; perpetuation; and, eventually, resolution of the process [6]. In this scenario, it was reasonable to think that interventions targeting not only lipid metabolism but also the inflammatory pathways would have an effect on atherogenesis [7,8]. However, there have been several failed attempts to prove that anti-inflammatory therapies may reduce CV events [9]. Among other reasons, these negative results may be partly explained by the fact that inflammatory pathways are also targeted by existing proven CV preventive therapies, such as statins and angiotensin-converting enzyme inhibitors. Recently, the Canakinumab Anti-Inflammatory Thrombosis Outcomes Study (CANTOS) trial showed that anti-inflammatory therapy targeted to inhibit IL-1 beta improves CV outcomes [10]. This large trial showed that canakinumab reduced the primary composite CV outcome, with a modest size effect. However, this effect was more pronounced among subjects with an increased persistent proinflammatory response, defined as a high C-reactive protein concentration. Following this line of evidence, several trials are currently underway to test other anti-inflammatory therapies targeting the innate and adaptive arms of the immune system in the prevention of ASCVD [9].

It should be emphasized that the involvement of inflammatory mechanisms does by no means exclude the important role of lipid metabolism in different steps of the atherosclerotic process. Several pieces of evidence demonstrate that lipids—in particular, cholesterol—are involved in the pathogenesis of atherosclerosis [11,12,13]. There is no doubt from epidemiological studies that deranged lipid metabolism, primarily low-density lipoprotein cholesterol (LDL) and other atherogenic lipoproteins, is a significant contributor to the development of atherosclerosis [14]. This evidence comes from data accumulated over decades resulting from genetic studies, Mendelian randomization studies, large prospective cohort studies, and randomized controlled trials (RCTs). Regarding the latter, different LDL cholesterol-lowering interventions have been shown in RCTs to contribute to the primary and secondary prevention of atherosclerotic CV events in different contexts and a wide array of underlying conditions [15,16]. There is general agreement that the magnitude of the effect of different LDL-lowering therapies is proportional to the decrease in LDL [17]. As such, the mainstream and simplified message is that the lower the LDL, the better the outcome. Furthermore, familial hypercholesterolemia is an illustrative and paradigmatic example of the role of cholesterol; subjects with this condition, even in the absence of any other CV risk factors or inflammatory diseases, develop a precocious and accelerated atherosclerotic process that leads to early CV events and CV death unless proper aggressive LDL-lowering therapy is introduced [18].

Both inflammation and lipid metabolism are closely linked, and their molecular pathways interact with each other in different tissues [19]. The atherosclerotic wall might be viewed as one of the body organs or tissues where the immune and metabolic systems convene. Relevant examples of organ/tissue meeting points for these two systems are the adipose tissue in obesity and the liver in non-alcoholic fatty liver disease (NAFLD). In both obesity and NAFLD, there is continuous crosstalk between the lipid metabolic and immune-inflammatory pathways in the adipose tissue and the liver, respectively. In fact, it has long been recognized that the metabolic and immune systems are among the most fundamental requirements for survival, and that this is why the respective pathways are closely related and evolutionarily preserved among species. As an illustrative example, species such as flies have a single functional organ, the fat body, that integrates the adipose tissue, the liver, and the hematopoietic system [19]. Moreover, it is well known that inflammatory mediators alone can trigger metabolic changes, but at the same time metabolic disturbances can induce inflammation [6]. All these concepts speak for an intimate relationship between the lipid and the inflammatory hypotheses. Consequently, we may state that, in atherosclerosis, both the immune and metabolic pathways are fundamental parts of the etiopathogenetic process of atherosclerotic disease. 

The lesson learned from the currently available literature is that, in terms of etiology, atherosclerosis stands as a multifactorial disease. As such, the evolution of the disease—and its increasing burden—is closely linked to diseases that relate to both metabolic and inflammatory changes in our population. Among those, we must consider an increased inflammatory innate immune response, including conditions related to aging, chronic kidney disease, obesity, hypertension, and type 2 diabetes. Therefore, all these factors, alone or in different proportions and combinations, are essential contributors nowadays that are closely linked to the appearance and progression of atherosclerosis globally.

From the pathogenetic point of view, the dissection of the different pathways involved in the atherosclerotic process is very relevant to draw a full picture of the pathogenesis and to identify potential new therapeutic targets. As for lipid-lowering interventions, promising further intervention immune-targeted strategies may contribute to the advancement of the prevention and treatment of ASCVD. However, relying on all the available background experimental and clinical evidence on the pathogenesis of atherosclerosis, the lipid hypothesis should not be considered alone or opposed to the immune-inflammatory hypothesis. In contrast, we propose that both explanations should be contemplated as non-mutually exclusive and consistently closely linked. As a concluding remark, we state hereby that atherosclerosis is the result of the complex crosstalk between the metabolism—in particular, the lipid metabolism—and the immune response. We therefore propose to name this the immune-metabolic hypothesis of the etiopathogenesis of atherosclerosis.
